# The role of viable but non-infectious developmental forms in chlamydial biology

**DOI:** 10.3389/fcimb.2014.00097

**Published:** 2014-07-24

**Authors:** Nicole Borel, Andreas Pospischil, Alan P. Hudson, Jan Rupp, Robert V. Schoborg

**Affiliations:** ^1^Department of Pathobiology, Institute of Veterinary Pathology, University of ZurichZurich, Switzerland; ^2^Department of Immunology and Microbiology, Wayne State University School of MedicineMichigan, MI, USA; ^3^Department of Molecular and Clinical Infectious Diseases, University Hospital Schleswig-Holstein/Campus LübeckLübeck, Germany; ^4^Department of Biomedical Sciences, Quillen College of Medicine, East Tennessee State UniversityJohnson City, TN, USA

**Keywords:** the chlamydial stress response, obligate intracellular, viable but non-infectious, aberrant bodies, chronic disease

Chlamydiae are both intriguing obligate intracellular Gram-negative bacteria and highly successful animal and human pathogens. Their unique biphasic developmental cycle is characterized by the infectious but metabolically less-active elementary body (EB), which initiates infection in a susceptible host cell, and the dividing, intracellular reticulate body (RB). The chlamydiae hijack host cells for their own purposes and, after infection and multiplication, emerge from the host cell and spread their hundreds of progeny EB to infect the next cell. Moreover, chlamydiae are able to survive in an alternative form named the aberrant body (AB) under stressful *in vitro* conditions. The AB state has been defined as the hallmark of persistence—which is herein defined as the viable but non-infectious state of the chlamydial developmental cycle. The key questions of whether this AB form occurs *in vivo* and if this alternative chlamydial form contributes to clinically significant chronic diseases processes such as inflammation, scarring, and fibrosis are a matter of debate in the chlamydial research community. These controversies led us to assemble a collection of Opinion, Hypothesis and Theory, Perspective, Review, and Primary Research Articles to shed light on recent research findings. This Research Topic showcases the “chlamydial persistent state” from meaning to mechanism on the cellular and human/animal host level.

The critical dissection of the word “persistence” by Bavoil (2014) points out that much more is hidden behind this term than the definition “to stand still permanently” in a susceptible host. For clinicians, this might represent a bacterial infection that: (i) has a subclinical course, (ii) can escape host immune responses; and (iii) is refractory to antibiotic treatment. The lack of specific diagnostic tests for such “invisible” infections might even complicate the definition and detection of these infections. Ongoing asymptomatic infections may even resemble colonization, as suggested by very recent data on chlamydial infections in the gastrointestinal tract. Notably, asymptomatic gastrointestinal chlamydial infections with recurrent chlamydial fecal shedding have been observed in the Veterinary Medical field for decades. For example, subclinical infections with *Chlamydia (C.) psittaci* in birds are frequent and these “latently” infected birds pose a significant zoonotic risk. Regardless, it is important for investigators in the Chlamydia field to come to agreement regarding the “definition” of what constitutes a persistent chlamydial infection.

The persistence phenotype *in vitro* may be linked to chronic disease processes *in vivo*, such as atherosclerosis. *C. pneumoniae* has been detected in atherosclerotic lesions of human patients by multiple methods and *C. pneumoniae*-induced disease progression has been demonstrated in rodent models—an association that propelled several antibiotic treatment trials. The opinion article by Campbell and Rosenfeld (2014) summarizes the outcome of these clinical trials and critically reviews their limitations. Unfortunately, the mixed results of these trials and the conclusion that anti-chlamydial antibiotics should not be recommended for treatment of patients with coronary heart disease and peripheral artery disease may have led to the erroneous assumption that *C. pneumoniae* did not play a role in the pathogenesis of atherosclerosis. The advanced nature of atherosclerotic lesions questions not only the effectiveness of antibiotic treatment to eradicate *C. pneumoniae* but also implicates how challenging the diagnosis of the agent may be. Puolakkainen (2013) discusses the difficulty in differentiating between acute and chronic infections due to the lack of reliable “persistence” biomarkers and commercially available tests. Whole proteome assays have been developed for research purposes but need careful validation on selected specimens from well-studied patient populations. The most desirable test to detect persistent infections would be serum-based, an optimistic goal that remains frustratingly out of reach without a better understanding of what persistence actually is and the molecular mechanisms that underly induction, maintenance, and recovery from this non-replicative state (Bavoil, 2014).

Azithromycin and tetracycline/doxycycline are considered the first-line antibiotics to treat genital chlamydial infection. *In vitro*, it has been shown that penicillin G exposure induces *C. trachomatis* persistent/aberrant forms. In culture, persistent organisms also resist killing by azithromycin. Because penicillins are commonly utilized to treat other bacterial infections, this may impact patients with concurrent asymptomatic chlamydial infections. Kintner et al. (2014) investigated if commonly prescribed beta-lactam antibiotics are able to induce *C. trachomatis* serovar E persistence/stress in culture. All penicillins tested induced viable but non-infectious chlamydial forms at clinically relevant concentrations, which might represent one mechanism by which chlamydiae resist antimicrobial therapy *in vivo*. In a related study, Ong et al. (2013) determined whether the *C. trachomatis* serine protease HtrA (CtHtrA) was required for recovery from penicillin-induced persistence/stress response. CtHtrA is essential for the replicative phase and the addition of a chemical inhibitor (JO146) of CtHtrA was lethal when added to the cultures at mid-replicative cycle. The same inhibitor prevented reversion and recovery from penicillin persistence, demonstrating the essential role of CtHtrA during recovery from stressful conditions. Not only is HtrA the first chlamydial gene shown to play a role in recovery from persistence, HtrA inhibitors might prove to be useful for eradicating both productively replicating and persistent chlamydiae.

Persistent infections have been described in a magnitude of experimental cell culture models—all of which involve applying some type of stressor to the chlamydial culture. The most well-described culture model of persistence is the IFN-γ-induced system. In human cells, IFN-γ exposure upregulates indoleamine 2,3-dioxygenase (IDO), decreases intracellular tryptophan (Trp), and induces chlamydial persistence. Chlamydial infectivity can be rescued by addition of exogenous Trp. Four manuscripts in this special issue explore novel aspects of IFN-stimulated persistence. Jerchel et al. (2014) explored *C. trachomatis* persistence and reactivation and host cytokine responses under normoxic and hypoxic conditions. Oxygen concentrations in the female urogenital tract are physiologically low and further diminished by inflammatory processes. This study demonstrated that hypoxia leads to reactivation of INF-γ-induced persistent *C. trachomatis* infections. Moreover, host immune signaling responses were diminished, as reflected by reduced activation of MAP-kinase p44/42 and reduced expression of pro-inflammatory cytokines IL-6 and IL-8. Recent sophisticated comparative evaluation of *Chlamydiales* proteomes for Trp content is reviewed by Bonner et al. (2014). Selection for higher-than-predicted (Up-Trp) Trp content, compared to lower-than-predicted (Down-Trp) content, is key for the persistent state and phylogenetically different for *Chlamydiaceae* compared to other chlamydiae families. Previous studies indicate that there is a significant difference in the capacity of ocular and genital *C. trachomatis* serovars to deal with tryptophan deprivation. Genital serovars encode a functional tryptophan synthase and can use indole to bypass INF-γ-induced tryptophan starvation. The perturbed vaginal microbiome as a source of indole *in vivo* is highlighted in the Hypothesis and Theory article by Aiyar et al. (2014). Indole has been found in vaginal secretions from patients with bacterial vaginosis and indole-producing bacteria have been isolated. Thus, when studying genital chlamydial infections in the future, it will be necessary to consider both the elicited host immune response and the local environmental conditions created by the host microbiome. Transferring such knowledge into the patient's situation is reflected in the article by Lewis et al. (2014). These authors were able to show persistent chlamydial growth forms in the human cervix by novel diagnostic approaches. Local IFN-γ levels corresponded to chlamydial growth pattern and morphology. This study may pave way to the establishment of potential biomarker panel for assessing the disease outcome in *C. trachomatis* infected women.

One ultrastructural feature of persistent chlamydiae observed in many cell culture models is production of abundant membrane vesicles (MVs), which are observed both within the inclusion and in the host cell cytoplasm. The potential role of these vesicles in host-pathogen interactions is explored by Frohlich et al. (2014). The generation of MVs is suggested to be an important mechanism for *C. trachomatis* intracellular survival of stress. MVs are not only present in primary human endocervical epithelial cells infected with *C. trachomatis*, but also in clinical specimens from infected patients (Lewis et al., 2014). The potential function and role of chlamydial MVs in cargo delivery, innate immune response modulation, exchange of genetic material, and intracellular survival under stress conditions calls for more research in this field.

The “chlamydial anomaly” refers to the observation that chlamydial species are sensitive to antibiotics that target the bacterial cell wall, such as penicillin, despite the fact that a functional cell wall has not been detected. This problem is featured in the research article by De Benedetti et al. (2014). The chlamydial genome contains a nearly complete cell wall precursor biosynthesis pathway. *Chlamydiaceae* genomes encode GlyA which in turn can serve as a source of D-alanine, as exemplified in *C. pneumoniae*. In view of this finding, future research might help to clarify the structure of cell wall precursor lipid II and the role of chlamydial penicillin-binding proteins in production of penicillin-induced aberrant chlamydial forms.

Finally, host-pathogen interplay between multiple pathogenic microorganisms, such as the porcine epidemic diarrhea virus (PEDV) co-infection model described in the last paper of this collection (Schoborg and Borel, 2014), might more appropriately reflect the *in vivo* situation than do artificially-induced cell culture models. In particular, this bacterial-viral co-infection has been shown to occur *in vivo*, can be mimicked in experimental porcine animal models and includes the local environmental conditions of the gut microbiome.

In summary, this collection highlights the recent findings in the field of the “persistent” chlamydial state, which might reflect persistence, re-occurrence, re-infection or colonization in an animal or human host. The collection also illuminates the multifaceted nature of chlamydial persistence and the complexity that results from the existence of different models and multiple factors involved. Moreover, many models have been developed from *in vitro* data and applicability to the *in vivo* situation is, as yet, undefined. As outlined by Bavoil (2014), our limited understanding of what persistence is needs further research in the future and will produce greater insight into the host-pathogen interaction.

## Conflict of interest statement

The authors declare that the research was conducted in the absence of any commercial or financial relationships that could be construed as a potential conflict of interest.
